# Prioritising Informed Health Choices Key Concepts for those impacted by cancer: a protocol

**DOI:** 10.12688/hrbopenres.13593.1

**Published:** 2022-08-04

**Authors:** Mengqi Li, Declan Devane, Claire Beecher, Austin G. Duffy, Caitriona Duggan, Maura Dowling, David Robert Grimes, Avril Kennan, Sarah McLoughlin, Allen Nsangi, Andrew D. Oxman, Robert O’Connor, Derek C. Stewart, Elaine Toomey, Marie Tierney

**Affiliations:** 1 School of Nursing & Midwifery, University of Galway, Galway, Ireland; 2 Health Research Board - Trials Methodology Research Network (HRB-TMRN), University of Galway, Galway, Ireland; 3 Evidence Synthesis Ireland and Cochrane Ireland, Galway, Ireland; 4 Department of Medical Oncology, Mater Misericordiae University Hospital, Dublin, Ireland; 5 Department of Oncology, Portiuncula University Hospital, Galway, Ireland; 6 School of Physical Sciences, Dublin City University, Dublin, Ireland; 7 Discipline of radiation therapy, Trinity College Dublin, Trinity Centre for Health Sciences, St. James's Hospital, Dublin, Ireland; 8 Health Research Charities Ireland (HRCI), Dublin, Ireland; 9 Informed Health Choices-Cancer, University of Galway, Galway, Ireland; 10 Department of Medicine, College of Health Sciences, Makerere University, Kampala, Uganda; 11 Centre for Epidemic Interventions Research, Norwegian Institute of Public Health, Oslo, Norway; 12 Irish Cancer Society, Dublin, Ireland; 13 College of Medicine, Nursing & Health Sciences, University of Galway, Galway, Ireland; 14 School of Allied Health, University of Limerick, Limerick, Ireland

**Keywords:** cancer; learning resources; informed choices; critical thinking

## Abstract

**Background: **Few areas of health have been as insidiously influenced by misinformation as cancer. Thus, interventions that can help people impacted by cancer reduce the extent to which they are victims of misinformation are necessary. The Informed Health Choices (IHC) initiative has developed Key Concepts that can be used in the development of interventions for evaluating the trustworthiness of claims about the effects of health treatments. We are developing an online education programme called Informed Health Choices-Cancer (IHC-C) based on the IHC Key Concepts. We will provide those impacted by cancer with the knowledge and skills necessary to think critically about the reliability of health information and claims and make informed choices.

**Methods:** We will establish a steering group (SG) of 12 key stakeholders, including oncology specialists and academics. In addition, we will establish a patient and public involvement (PPI) panel of 20 people impacted by cancer. After training the members on the Key Concepts and the prioritisation process, we will conduct a two-round prioritisation process. In the first round, 12 SG members and four PPI panel members will prioritise Key Concepts for inclusion. In the second round, the remaining 16 PPI members will undertake the prioritisation based on the prioritised Key Concepts from the first round. Participants in both rounds will use a structured judgement form to rate the importance of the Key Concepts for inclusion in the online IHC-C programme. A consensus meeting will be held, where members will reach a consensus on the Key Concepts to be included and rank the order in which the prioritised Key Concepts will be addressed in the IHC-C programme.

**Conclusions: **At the end of this process, we will identify which Key Concepts should be included and the order in which they should be addressed in the IHC-C programme.

## Introduction

Cancer is the first or second leading cause of death in more than 100 countries in the world ^1, 2^, having overtaken high mortality chronic diseases such as cardiovascular diseases in many countries ^3^. Cancer also contributes to substantial and persistent disease burdens on patients and their families, such as financial and psychological burdens. According to the International Agency for Research on Cancer (IARC), the global cancer burden is expected to reach 28.4 million cases by 2040, a 47% increase from 2020 ^2, 4^.

The growing incidence of cancer, the lack of unified treatments, and the fact that many types of cancer are not entirely curable can lead to a fear of cancer disproportionate to its severity ^5, 6^. People with a cancer diagnosis will often seek health information to inform their decisions about treatment options ^7, 8^. However, much of the information available is unreliable. Reports suggest that more than half of the most widely shared cancer articles on social media are discredited health claims ^9, 10^. Furthermore, people’s ability to make informed choices about their health has been undermined by excessive information, which has generated a crisis of knowledge that is described as one of the most significant risks to health and health care ^11^.

People are confronted almost daily with information or claims on social media about the effectiveness of specific treatments that may help maintain health or treat ill-health ^12, 13^. However, many of these claims are biased, inaccurate, or unsubstantiated, whether well-intentioned or motivated by commercial or other interests ^11, 14^. Unreliable information and claims about treatments may lead to inadequate or excessive health treatments, which may cause harm for the individual ^11^ and waste limited resources ^12^. Lack of ability to judge the reliability of health information at the individual level can lead to delays in standard treatment and unavoidable harm, including death, while at the collective public level, it may alter the attitudes and behaviours of people, including policies on public health issues.

Few areas of health are as influenced by misinformation as cancer ^10^. A recent study by Johnson *et al.* ^9^ found that 33% of articles on the four most common cancers on Facebook and other social media sites contained misinformation. Among those containing misinformation, 77% contained harmful information ^9^. The often-long course of cancer treatment, the different stages of some types of cancer and the potential treatment options may account for the proliferation of misinformation in the cancer field.

Reliable information can help reduce uncertainty in treatment decisions for those impacted by cancer and help alleviate anxiety and depression ^9, 15^, thus facilitating their eventual recovery processes ^6^. While it may not be possible to change the volume of information available, improving critical thinking about information and claims about the effects of treatments may help people identify and act on trustworthy claims and not act on those that are not trustworthy.

When a person or their loved ones are diagnosed with an illness such as cancer, the illness may become a turning point and dramatically influence their attitudes toward treatments and decisions ^16^. Research has found that because of the eagerness to seek therapeutic results, people with a cancer diagnosis can place faith in fundamentally flawed therapies ^17^, which can lead to unnecessary human suffering and an up to five times higher mortality rate depending on the cancer type ^18^. Therefore, it is essential that people impacted by cancer are helped to protect themselves from being misled, intentionally or unintentionally, into making decisions about their health based on unreliable information and health claims.

An international collaboration of researchers led by Oxman *et al.* has developed the Informed Health Choices (IHC) initiative ^19^. The IHC initiative is a structured, evidence-based programme that aims to enhance informed decision-making in health by providing people with the skills and knowledge necessary to understand and critically appraise the trustworthiness of a treatment claim.

The core of the IHC project is a suite of 49 Key Concepts that provide principles for evaluating the trustworthiness of treatment claims, comparisons, and health choices ^20^. They empower people to recognise when health claims (the effects of doing or not doing something) are made, assess the trustworthiness of the evidence used to support the claims, and make well-informed choices when choosing alternative courses of action ^21– 24^. The IHC Key Concepts Framework draws on existing theoretical frameworks within the fields of health literacy and critical thinking. In practical terms, the IHC framework provides a means of going beyond the delivery of information to developing critical thinking skills. It supports critical thinking about the choices people make by helping them recognise and act on reliable information and, conversely, not act on misinformation. The Key Concepts have been adapted and used in fields such as agriculture, education and policing ^25^.

The effectiveness of a primary school intervention based on the IHC Key Concepts has been evaluated in a large cluster-randomised trial in Uganda ^26^. The authors concluded that the programme improves the students’ and their parents’ ability to assess health claims. The improvements were shown to have been sustained over a one-year follow-up ^27^.

Building on the IHC Key Concepts initiative, we will develop an online education programme called the Informed Health Choices-Cancer (IHC-C) programme to provide those impacted by cancer with the skills and knowledge necessary to think critically about the reliability of treatment claims and make informed choices. The programme will be developed in two work packages (WPs). WP1 will adapt the protocol used by Oxman *et al.* for lower secondary schools in East Africa to prioritise IHC Key Concepts to be included in the IHC-C programme and identify the order in which they will be addressed within the programme ^28^. WP2 will develop an online education programme based on the Key Concepts prioritised and ordered in WP1 and explicitly tailor these to a cancer population. This protocol details the methods of WP1.

### Aim

This protocol describes the methods used to identify the IHC Key Concepts to be included in the IHC-Cancer programme and prioritise the order in which the Key Concepts will be addressed in the programme.

## Protocol

### Ethical approval and consent

The ethical approval for this study was granted by the University of Galway Research Ethics Committee (reference: 2022.03.005). Informed consent will be sought from all participants prior to their participation in this study.

**Figure 1.**
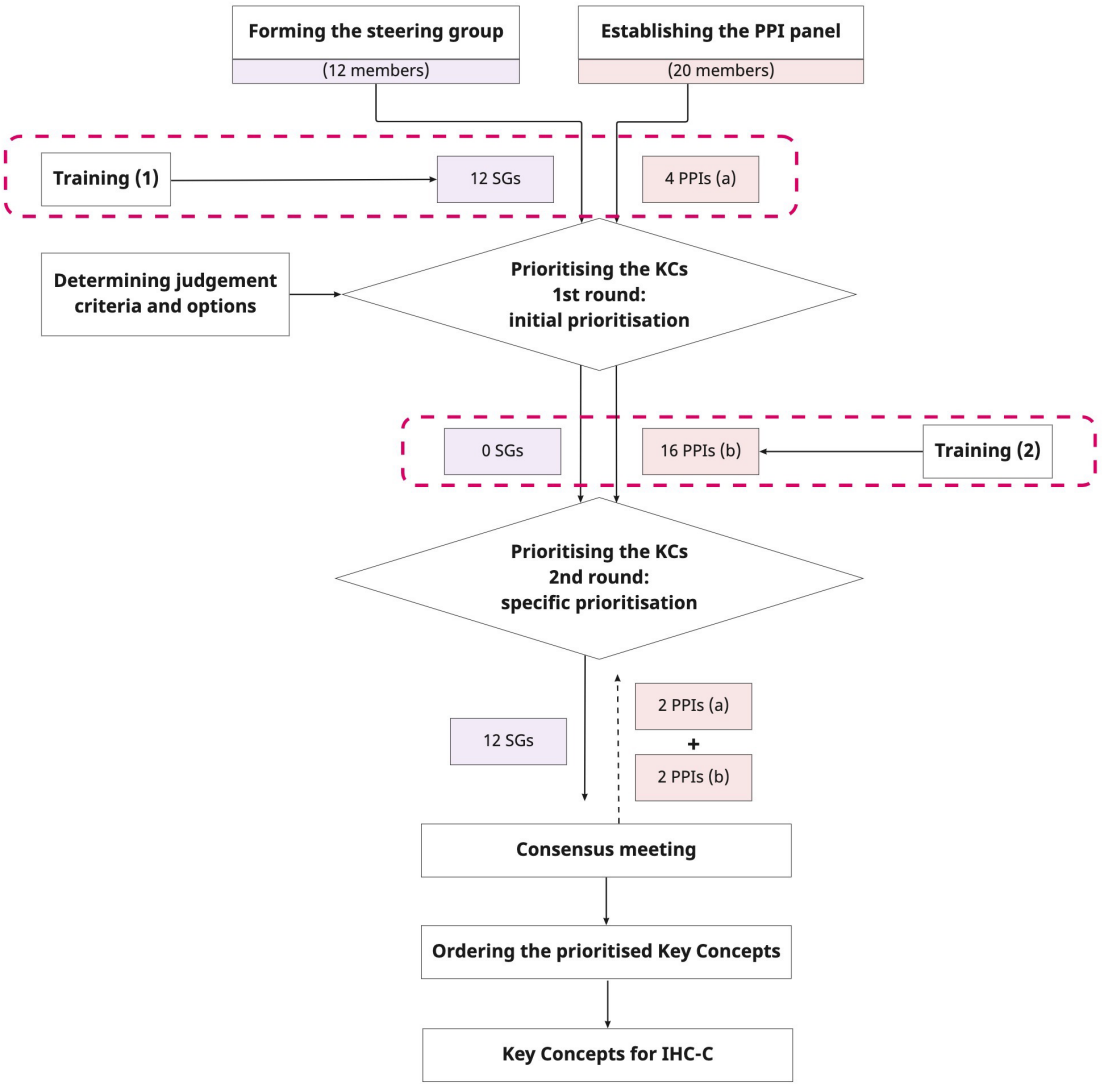
Prioritisation process for the IHC-C Key Concepts. *( **a**) ( **b**): The 20 PPI members will be divided into two groups for the two rounds of prioritisation. Group ( **a**) will include four PPI partners who will participate in the first round of prioritisation, and group ( **b**) will include 16 PPI partners who will participate in the second round of prioritisation. *PPI: Patients and Public Involvement; PPIs: Patients and Public Involvement members; SGs: steering group members; KCs: Key Concepts; IHC-C: Informed Health Choices-Cancer.

This protocol is reported according to the *Reporting Guideline for Priority Setting of Health Research (REPRISE)* ^29^. The prioritisation process consists of the following steps (see Figure 1):

1. Forming the steering group
2. Establishing a patient and public involvement panel
3. Training members of the steering group and patients and public involvement panel
4. Prioritising the IHC Key Concepts
5. Collating and analysing feedback, ordering the prioritised Key Concepts, and reaching a final consensus

### 1. Forming the steering group

We will establish a Steering Group (SG) to ensure that key stakeholders will guide and shape the development of the IHC-C education programme. We will introduce the IHC-C programme to stakeholders (people impacted by cancer, medical oncologists, cancer nurses, cancer researchers, methodology researchers, experts from the IHC Key Concepts initiative and experienced educationalists). We will invite those individuals interested in developing this programme to join the IHC-C project.

### 2. Establishing a patient and public involvement panel

The involvement of people impacted by cancer is central to developing an IHC-C programme grounded in the lived experiences of those impacted by cancer ^30– 32^. We will establish a Patient and Public Involvement (PPI) panel comprising approximately 20 people impacted by cancer, defined as:

- people diagnosed with any type of cancer,
- survivors of any type of cancer,
- informal caregivers of people with cancer,
- loved ones of people with cancer (family, friends, or others who care about those with cancer).

We will recruit PPI panel members based on the eligibility criteria and through three channels to ensure that a diversity of people impacted by cancer (e.g., different ages, ethnicity, level of education, cancer types etc.) are included ^33^.

We will initially undertake a social media (Instagram, Facebook, and Twitter) campaign to recruit people impacted by cancer for our PPI panel. We recognise that this recruitment process may not enable the diversity of panel participants that we seek. Should that be the case, we will seek to recruit panel participants via the Irish Cancer Society Daffodil Centres, local cancer support centres and oncology outpatient clinics (Appendix 1).

In all recruitment channels, brief study information will be made publicly available, and potential participants will be invited to contact the research team to receive further information about the study. This will help limit any perception of external pressures from the research team or those supporting the research team on the potential participants to engage with the study. Potential participants who provide their contact information to the research team will be provided with an information leaflet, consent form and an expression of interest form. While we plan to recruit 20 people impacted by cancer, we recognise that not all who agree to take part will do so or be able to do so. Therefore, we will recruit up to 22 participants.

### 3. Training members of the steering group and patients and public involvement panel

Once the SG and PPI panel members have been identified, we will provide all members with both offline and online training. We will provide participants with preparatory reading materials by email or hard copy at least two weeks before the online training. All participants will be asked to familiarise themselves independently with the materials before the online training. Any questions participants have will be answered by the research team in advance or brought for discussion during the online session. The online training will be held in the form of separate but almost identical group meetings for each round of prioritisation that focuses on:

***3.1 Introduction to the IHC initiative.*** We will briefly introduce the IHC project, including the problem it is trying to address and the solution the IHC-C seeks to provide.

***3.2 Introduction to the IHC-C programme.*** We will familiarise all members with the details of the IHC-C programme, including the rationale for the project, purpose and processes involved.

***3.3 Introduction to the IHC Key Concepts.*** We will explain the background of the formation and the IHC Key Concepts. We will provide participants with a list of all 49 Key Concepts and explanatory information (the list of all 49 key concepts can be found in the extended data). The 49 IHC Key Concepts are divided into three sections i.e., claims, comparisons, and choices ^21, 22^. An example of an IHC Key Concept is "it should not be assumed that treatments are safe or effective - or that they are not". We will select two exemplar Key Concepts from each section to illustrate the Key Concepts. We will discuss each of these six exemplar concepts in detail, including the concept, its explanation with accompanying examples and the implications arising from the concept. We anticipate that this approach will help participants understand the principle and structure of the Key Concepts in a way that will enable them to review the 49 concepts. This is in line with evidence suggesting that adults can develop an understanding of the content they are interested in through independent study ^34^. We will check with participants that this is the case and, if not, introduce additional education as required. We will make the complete resource of 49 Key Concepts and all the materials we used to explain the exemplar Key Concepts available to all members who participate in the prioritisation.

***3.4 Prioritisation process.*** We will explain the purpose of the prioritisation process and the steps involved, including what we are asking participants to do.

In addition to encouraging participants to ask questions or make any comments, at any point during the training, we will hold a separate question and answer session at the end of the training.

All participants will also be informed that the online training meeting will last up to 2 hours and will be recorded to allow members to watch back if they still have questions about any part after the training.

### 4. Prioritising the IHC Key Concepts

***4.1 Determining judgement criteria for Key Concepts prioritisation.*** The SG and PPI panel members will review the IHC Key Concepts and prioritise those they feel should be included in the IHC-C education programme. This will be done using an online judgement form via QuestionPro software ^35^, which asks participants to review each of the 49 Key Concepts and judge if they are (1) easy; (2) relevant; (3) how likely is it that people are already aware of this concept; and (4) how helpful the Key Concept is in supporting people to assess treatment claims or make well-informed choices; and (5) whether the concept should be included in the IHC-C education programme (see Table 1 and Appendix 2). In addition, participants will be able to leave free text comments on each Key Concept and on the overall Key Concepts if they wish.

**Table 1.** Judgement form.

**Concepts**	**Criteria**				**Judgements**	**Comments**
	1. How easy is it to understand this concept?	2. How relevant (i.e., of current significance or importance) do you think this concept is?	3. How likely is it that people are already aware of this concept?	4. How helpful do you think this concept is in supporting people to assess treatment claims or make well-informed choices?	Do you think this concept should be included in an education programme to help people impacted by cancer think critically about the reliability of a treatment claim or make well-informed choices?	
**Concept 1**	☐ very easy ☐ easy ☐ uncertain ☐ hard ☐ very hard	☐ very relevant ☐ relevant ☐ uncertain ☐ irrelevant ☐ very irrelevant	☐ very likely ☐ likely ☐ uncertain ☐ unlikely ☐ very unlikely	☐ very helpful ☐ helpful ☐ uncertain ☐ unhelpful ☐ very unhelpful	☐ yes ☐ no (If no, please state why: ☐language; ☐readable; ☐design of instructions; others please specify: ____________________)	
**Concept 2**						
**Concept 3**						

***4.2 Prioritising the Key Concepts.*** Participants will be invited to prioritise the Key Concepts for inclusion in the IHC-C education programme in two rounds. Both rounds of prioritisation aim to identify and prioritise which of the Key Concepts should be included in the programme. We will invite the 12 SG members and an additional four PPI panel members to undertake initial prioritisation using the judgement form (described in 4.1). The second round of prioritisation will be conducted with sixteen PPI panel members who will use the same process and criteria used in the first round.

The prioritisation data collected will be extracted into secure data files, and all data will be stored in an organised file system on a secure network. We will use SPSS 26.0 statistical software to analyse the data using frequencies, percentages, medians (quartiles), and means (SDs) as appropriate.

### 5. Collating and analysing feedback, ordering the prioritised Key Concepts, and reaching a final consensus

After receiving responses and feedback from the two rounds of prioritisation, the research team will collate the feedback and develop the first draft of the IHC-C Key Concepts, which will be sent to the 12 SG members and four PPI panel members, of which two will be from the first-round prioritisation, and two will be from the second-round prioritisation. Subsequently, a consensus meeting will be held with these 16 members.

Participants at the consensus meeting will be presented with Key Concepts that were included and excluded after the two rounds of prioritisation. Participants will be invited to share their thoughts, opinions, and suggestions on the prioritised Key Concepts. Discussion will be invited, and the final set of Key Concepts for inclusion in the education programme will be agreed.

We will then ask participants to rank the order in which the Key Concepts should be presented within the educational programme. This is done on the assumption that while all 49 Key Concepts are equally important in improving people’s critical thinking, participants will likely have a preference for the order in which the Key Concepts are introduced within the education programme.

## Discussion

Empowering people impacted by cancer with the knowledge and skills necessary to think critically about the reliability of health information and claims and make informed choices is important. In WP1 of IHC-C programme, based on the IHC Key Concepts, members of the steering group and PPI panel will complete a two-round prioritisation, and through a joint consensus meeting, we will determine the prioritised and ranked Key Concepts resource for managing misinformation in cancer, which will also be the basis of the IHC-C intervention programme.

### Dissemination

We aim to disseminate the findings of this study via relevant meetings, conferences and peer-reviewed academic journals.

### Study status

Recruitment for the PPI panel members commenced in July 2022.

## Data availability

### Underlying data

No underlying data are associated with this article.

### Extended data

Zenodo: Prioritising Informed Health Choices Key Concepts for those impacted by cancer: a protocol_Appendix. https://doi.org/10.5281/zenodo.6817186 ^33^

This project contains the following files:

- Appendix 1: patient and public involvement (PPI) panel recruitment.pdf
- Appendix 2: Informed Health Choices-Cancer judgement form.pdf

Zenodo: Key Concepts for assessing claims about treatment effects and making well-informed treatment choices (Version 2022). https://doi.org/10.5281/zenodo.6611931 ^36^

Data are available under the terms of the Creative Commons Attribution 4.0 International license (CC-BY 4.0).
