# Supplementary material for: Prioritising Informed Health Choices Key Concepts for those impacted by cancer: a protocol
Source: HRB Open Res. 2022 Aug 4;5:55. [Version 1] doi: 10.12688/hrbopenres.13593.1 (PMC10518847; doi:10.12688/hrbopenres.13593.1)
Supplement: Peer review history

# Reviewer report: Reviewer response for version 1

**DOI:** 10.21956/hrbopenres.14849.r35897

**Published:** 2023-09-11

**Authors:** Sibel Saya

**Affiliations:** 1 The University of Melbourne, Melbourne, Victoria, Australia

This is an important program of work whose rationale is well described and clear. The protocol for the development of the IHC for cancer is fairly thoroughly described and I would only have a few comments on clarity.

 It would be useful as a reader to understand earlier in the paper or have some concrete examples of the IHC Key Concepts. This would help orient the reader as to the specifics of what the IHC-C will cover and what the PPI panel will review.

 Section 3.3 - it would be useful to understand more about how the participants' understanding of the Key Concepts will be 'checked' and what the additional education will be if this is deemed not sufficient.

 Section 4.2 - I don't feel it is clear about what criteria/cut-offs will be used to determine whether a Key Concept will be included in the IHC-C or not. There is only one sentence on statistical analysis, which is a bit vague. How much will this quantitative data determine inclusion versus the qualitative free text comments? I think this could use a lot more detail.

 I feel the Discussion could provide a bit more detail, in particular on the IHC-C intervention program and the Key Concept resource.

Is the study design appropriate for the research question?

Yes

Is the rationale for, and objectives of, the study clearly described?

Yes

Are sufficient details of the methods provided to allow replication by others?

Yes

Are the datasets clearly presented in a useable and accessible format?

Yes

Reviewer Expertise:

Epidemiology, genetic counselling, health services research, decision-making, cancer screening

I confirm that I have read this submission and believe that I have an appropriate level of expertise to confirm that it is of an acceptable scientific standard.

# Reviewer report: Reviewer response for version 1

**DOI:** 10.21956/hrbopenres.14849.r32631

**Published:** 2022-08-26

**Authors:** Daniel Semakula

**Affiliations:** 1 THRiVE, c/o School of Medicine, College of Health Sciences, Makerere University, Kampala, Uganda; 2 Johns Hopkins University School of Medicine, Baltimore, MD, USA

The authors intend to set priorities for a set of Key concepts for inclusion in an educational intervention for enabling cancer patients to assess the trustworthiness of claims about the effects of treatments. They plan to do this by setting up panels of key stakeholders, training them about the key concepts, and working with them to set priorities for the key concepts to be included.

 Overall, I have found this manuscript to have sufficient details. The motivation for the study is well articulated, and the aims and methods are generally well described. Below, I have highlighted some questions and comments about areas that I think could be improved upon. These comments should, at least, be interpreted as points of reflection.

 The main question I would like the authors to reflect on is: to what extent does the whole priority setting process as envisioned lend itself to elements of successful priority setting such as those described in:

 Sibbald, S.L., Singer, P.A., Upshur, R. et al. Priority setting: what constitutes success? A conceptual framework for successful priority setting. BMC Health Serv Res 9, 43 (2009). https://doi.org/10.1186/1472-6963-9-43 ^1^. 

 More specific comments and questions are highlighted below:

**1. Forming the steering group and PPI**

 Inclusion of participants from marginalized groups/neighbourhood: I have noted the authors’ considerations for diversity and inclusion in the selection of participants for the PPI panels. However, I wonder if, besides age, sex, education, cancer type, and gender, other social determinants of health such as neighbourhood and socioeconomic status were considered in populating these groups. In addition, I would consider disability status. These variables could have a greater influence on access to information for decision-making, educational economic opportunities as well as health outcomes, and overall well-being. In fact, one could argue that a lack of consideration for housing status/neighbourhood or disability status, for example, could contribute to inequity in public engagement. To the extent that it is possible, it might be worth reflecting on these, as social justice and equity matters pertaining to public and patient involvement in research.

**3. Training members of the steering group and patients and public involvement pane**

 The IHC concepts are likely to be new to the participants. How will you assess the understanding of information among panelists before they prioritise them? This is especially important as there is likely to be little if any qualitative data collected to explain how people prioritized the concepts.

**4. Prioritising the IHC Key Concepts**

 Could you please elaborate on the techniques for consensus building that you will be using?

 Relatedly, how will you address disagreements and dissent?

 From previous experience, an understanding of some of the key concepts might be required in order to ensure a better understanding of others. Some build on the knowledge of others. How do you plan to incorporate this in your priority setting exercise especially? Is there likely to be a conflict between the conflicts prioritized by participants and those which from previous research are hypothesized to be required as a foundation for the understanding of others?

 “Participants will be invited to prioritise the Key Concepts for inclusion in the IHC-C education programme in two rounds.” How have you determined that two rounds will be enough? The principles of public engagement require that the right participants, enough participants are engaged and that participants should be engaged enough. How have you determined that your current numbers will be sufficient? What mechanisms have you instituted to ensure that if the numbers are not sufficient you will add more? How will you determine if the quality of engagement was sufficient? You might find some ideas from https://www.hra.nhs.uk/planning-and-improving-research/best-practice/public-involvement/. Lastly, one of the key challenges of public involvement is evaluation. There is evidence to suggest that very few research undertakings evaluate their patient and public engagement activities. How will you evaluate the success of the engagement process and the priority-setting exercise?

**Table 1. Judgment form**

 Judgments: I understand the need for a Yes/No variable/question in making final judgments: “Do you think this concept should be included in an education programme to help people impacted by cancer think critically about the reliability of a treatment claim or make well-informed choices?” With this said, have you reflected on whether you might get better data with Likert-type scale data for the judgment question?

**Dissemination plan**

 Noted plans to disseminate findings through journals and conferences. Have you considered other ways of dissemination to other relevant stakeholders who may not have ready access to conferences and journals? How will you ensure that you address any equity and social justice issues that could potentially arise in the dissemination process?

Is the study design appropriate for the research question?

Yes

Is the rationale for, and objectives of, the study clearly described?

Yes

Are sufficient details of the methods provided to allow replication by others?

Yes

Are the datasets clearly presented in a useable and accessible format?

Not applicable

Reviewer Expertise:

Health systems/health services research, evidence informed decision making, public health and policy, epidemiology, biostatistics, research methods

I confirm that I have read this submission and believe that I have an appropriate level of expertise to confirm that it is of an acceptable scientific standard.

## References

[1] : Priority setting: what constitutes success? A conceptual framework for successful priority setting. *BMC Health Serv Res* .2009;9: 10.1186/1472-6963-9-43 43 10.1186/1472-6963-9-43 19265518PMC2655292
